# Perceived Vulnerability to Disease, Knowledge and Preventive Behavior Related to COVID-19 in Farsi and Arabic Speaking Refugees

**DOI:** 10.1007/s10903-021-01322-4

**Published:** 2021-12-10

**Authors:** Schahryar Kananian, Samar Al-Sari, Ulrich Stangier

**Affiliations:** Department for Clinical Psychology, Goethe University Frankfurt, Varrentrappstr. 40-42, 60486 Frankfurt, Germany

**Keywords:** Contamination, Refugees, COVID-19, Pandemic, Perceived vulnerability to disease

## Abstract

In the face of the worldwide COVIV-19 pandemic, refugees represent a particularly vulnerable group with respect to access to health care and information regarding preventive behavior. In an online survey the Perceived Vulnerability to Disease Scale, self-reported changes in preventive and risk behaviors, knowledge about COVID-19, and psychopathological symptoms (PHQ-4) were assessed. The convenience sample consisted of *n* = 76 refugees (*n* = 45 Arabic speaking, *n* = 31 Farsi speaking refugees) and *n* = 76 German controls matched with respect to age and sex. Refugees reported a significantly larger fear of infection, significantly less knowledge about COVID-19, and a higher frequency of maladaptive behavior, as compared to the control group. This study shows that refugees are more vulnerable to fear of infection and maladaptive behaviors than controls. Culturally adapted, easily accessible education about COVID-19 may be beneficial in improving knowledge and preventive behaviors related to COVID-19.

## Introduction

In September 2021, more than 215 million people worldwide have been infected with the virus SARS-CoV-2, with nearly 5 million deaths [1]. Given the lack of vaccination and treatment, international experts agree that prevention is the preferred way to contain the pandemic. Isolation, quarantine, social distancing, and community containment measures are curial for coping with the current restrictions. At the same time, there are more than 41 million internally displaced people and 25 million displaced refugees globally, living in large-scale refugee camps or being gathered in boats or busses during the flight [2]. The extremely densely populated conditions and limited access to sanitation systems and health facilities place refugees at particularly high risk to get infected.

Similarly, also refugees who reached host countries in Western countries often live in housing conditions that impede social distancing and increase the risk of infection with COVID-19, e.g. shared rooms and sanitary facilities. Furthermore, in case of infection, refugees are threatened by income loss, health-care insecurity, and the ramifications that come with postponement of decisions on their legal status or reduction of employment, legal, and administrative services [3]. In addition, refugees have restricted access to culturally and linguistically appropriate information about the spread of COVID-19, and how to protect oneself and others [4]. Thus, refugees are especially exposed to unreliable health information in social media or on daily front page headlines in the popular press [2]. However, the influence of information and knowledge on preventive behavior is essential [5].

Finally, cultural influences on coping behavior may further increase the risk of infection. For instance, cultural differences in the Hofstede Power Distance and Masculinity may predict the compliance with campaigns to improve infection control [6]. Interestingly, an international study on the impact of culture on social distancing found that Uncertainty Avoidance Index significantly predicted social gathering, with higher uncertainty avoidance being associated with lower social gathering [6].

A psychological theory to explain individual differences in the emotional and behavioral reactions to pandemics is the concept of behavioral immune system. This theory postulates that coping responses to pandemics are largely influenced by evolutionary determined patterns of disgust, avoidance and fears related to the source of infection [7, 8]. According to this theory, perceived vulnerability to disease (PVD; 9) may determine preventive behaviors, such as avoiding contact with infected individuals. There is empirical evidence that disgust sensitivity may influence avoidance of contamination [10–12]. From an evolutionary perspective, it seems disgust sensitivity can also enhance positive preventive behavior [12, 13]. Deacon and Olatunji [13, 14] showed that disgust sensitivity could predict emotional and behavioral reactions towards infection-related stimuli. In a recent study, we found that disgust sensitivity and fear of infection, as measured by the PVD scale, were significantly associated with increased social distancing, using masks and disinfectants defectants, and hand washing [15].

With regard to refugees, cultural factors might moderate the influence of perceived vulnerability to disease. For example, Skolnick and Dzokoto [16] found a higher PVD in Ghanaian participants compared to American participants. Moreover, disgust connoting contamination also produced larger cross-cultural effect sizes than other types of disgust, such as moral disgust. In Iran, discrepancies between men and women regarding PVD were found that might be bound to culturally shaped gender role ideals, such as women being more susceptible to diseases then men [17]. Oaten and colleagues [18] argued that perceived vulnerability to infections might be higher in regions where infectious diseases are more prevalent. In Afghanistan or Syria, rates of several infectious diseases, such as malaria or tuberculosis, are significantly increased [19, 20].

The present online survey was conducted during the lockdown in Germany (April 2020) and aimed at comparing perceived vulnerability to disease, knowledge and behaviors related to COVID-19 pandemic and emotional distress in refugees and a matched German control group. We examined the hypothesis whether refugees from Arabic speaking countries (Syria and Iraq) and Farsi speaking countries (Afghanistan and Iran) show a higher perceived vulnerability to disease than controls from Germany. In addition, we expected that refugees have less knowledge about COVID-19 pandemic, show less preventive and more risk behaviors in response to the lockdown.

## Methods

### Participants and Procedure

This study was based on an online-survey created with EFS Survey. The Data was collected between March 24 and April 28, 2020. The survey was assessed at a relatively early state of the pandemic, when first social restrictions were announced. The study was approved by the research ethics board of [blinded out for review]. Participants were recruited via flyers in refugee camps, social workers, local newspapers, TV, and the counseling center for refugees at [blinded out for review]. No payment was given for participation.

The sociodemographic characteristics are presented in Table 1. As intended through the matching procedure, the control sample of German citizens did not differ significantly from the refugee with regard to age, sex, or housing. However, there was a significant difference in education, *p* = 0.002, *T* = 1.18, *df* = 150, indicating that refugees had a lower level of education (see Table 1). The within group comparison for the refugees’ sample did not reveal any differences between Farsi and Arabic speaking refugees for the educational level (*p* = 0.652, *T* = − 4.53, *df* = 74).

**Table 1:** Sociodemographic characteristics of participants

	Controls		Refugees total sample		Farsi		Arabic	
	*n*	%	*n*	%	%	%	*n*	%
Sex	76	100	76	100	31	100	45	100
Male	26	34.2	26	34.2	11	35.5	15	33.3
Female	50	65.8	50	65.8	20	64.5	30	66.7

	*M*	SD	*M*	SD	*M*	SD	*M*	SD
Age in Years	39.1	13,2	37.5	9.5	40,6	10	36,5	9,3

### Measures

*Perceived vulnerability to disease.* Worries about contagious diseases were assessed using the Perceived vulnerability to disease (PVD;10). The PVD is a self-report questionnaire and consists of 28 items. Items are scored on a 7-point Likert scale, ranging from 1 (*strongly disagree*) to 7 (*strongly agree*). The PVD comprises two subscales: perceived infectability (PI; susceptibility to disease) and Germ Aversion (GA; emotional discomfort in certain contexts). The PVD has performed well on tests of reliability and validity [9, 21–23]. In the present study, two items were excluded which appeared to be no more appropriate to contemporary life conditions (Item 4: *write with a pencil someone else has obviously chewed on*; Item 15: *avoid using public telephones because of the risk that I may catch something from the previous use*). The Farsi version of the PVD has been validated (1818). The Arabic version was provided by the authors via the standard procedure in line with recommendations by Van Ommeren [24] (Table 2).

**Table 2:** Means and standard deviations of COVID-19 behavior checklist, knowledge on COVID-19, and patient health questionnaire (PHQ-4)

Measure	Refugees		Controls		ANOVA
	*M*	SD	*M*	SD	(df = 1, *p*)
PVD	3.85	*1.32*	3.2	*1.01*	0.003
CBC risk	− 1.12	*1.43*	− 1.59	− *1.21*	0.033
CBC adaptive	0.10	*0.96*	0.64	*0.72*	0.001
CBC preventive	0.059	*1.73*	1.83	*1.1*	0.001
CKQ	12.66	*4.32*	17.72	*3.02*	0.001
PHQ-r	2.01	*0.81*	1.87	*0.92*	0.204

*PVD* perveived vulnerability to disease, *CBC* COVID-19 behavior checklist, *CKQ* knowledge about COVID-19, *PHQ-4* patient health questionnaire

In line with previous studies [21], the internal consistency of the germ aversion subscale in the present study was very low (Cronbach’s α = 0.35) and of the subscale perceived infectability (Cronbach’s α = 0.72) only moderate. Since internal consistency of the total score was on a satisfying level (Cronbach’s α = 0.78), we used only the total score.

*Depressive Symptoms* Symptoms of anxiety and depression were assessed with the Patient Health Questionnaire (PHQ-4; 26). This instrument consists of 4 items that refer to the DSM-5 criteria for Major Depression. Items are measured on a 4-point Likert scale ranging from 0 (*not at all*) to 3 (*nearly every day*). The instrument has been validated in various samples [25, 26]. The PHQ-9 has already been translated and validated in Farsi with a high test–retest reliability and a good construct validity [27]. For this study the authors translated and back translated the PHQ-4 to Farsi and Arabic. This was done in line with the stand procedure for the translation of diagnostic instruments [24]. Internal consistency for the current study was excellent for the German and Farsi (Cronbach's α = 0.89), but only sufficient for the Arabic version (Cronbach’s α = 0.68).

*Knowledge about COVID-19.* The authors developed a questionnaire as a multiple-choice test (*COVID-19 Knowledge Questionnaire *[*CKQ*]) mainly using the frequently asked questions posted on the website of the World Health Organization [1] and German Federal Ministry of Health [28]. We replaced the term COVID-19 by “corona infection” as this was more used in daily life in Germany. The CKQ consisted of seven questions, one question each on disease epidemiology (3 correct answers out of 6), symptoms (5/10), incubation period (1/3), mode of transmission (5/8), fatality rate (1/4), risk factors (4/6) and preventive strategies (4/7). For face validity, three medical doctors reviewed the final version. The general score was the percentage of correct answers, averaged over all seven questions. High Scores indicate a good knowledge according to the WHO, as lower scores indicate misinformation. Farsi and Arabic versions were provided according to the translation procedure by van Ommeren et al. [24].

*COVID-19 Behavior Checklist *(*CBC*) Behavioral changes during lockdown were assessed with a checklist of behaviors which was developed by the authors. The items referred to commonly discussed practices of hygiene (3 items), social activities associated with physical contact (3 items) or social distance (1 item), and health-related activities (4 items). Behavioral changes were assessed on a 7-point Likert scale, ranging from -3 (*much less)* to + 3 (*much more frequent*), with 0 representing “*unchanged”*. The questionnaire consisted of three subscales: (1) Risk Behavior, (2) Preventive Behavior, and (3) adaptive behavior. The CBC was translated in Farsi and Arabic according to the suggested procedure by van Ommeren et al. [24].

### Data Analysis

The German sample was matched on age and sex, using the propensity score matching module of R [29]. For the following analyses, SPSS-27 was used. Group differences between German and Farsi/Arabic speaking participants with regard to CKQ, PVD, PHQ-4, and behavioral changes were tested with a MANOVA. In addition, to compare PVD for both groups we calculated an ANCOVA with CKQ as a covariate to control for possible confounding of PVD and CKQ. Linear regression analyses were conducted with CKQ and PVD as independent and CBC and emotional distress dependent variable.

## Results

A significant overall group (refugees vs. controls) effect resulting in a Pillai’s trace value of 0.972, *F*(6, 145) = 850.92, *p* < 0.001, indicated that refugees differed significantly with respect to the dependent variables. Univariate analyses showed that significant differences were observed in PVD (F = 8.96, *p* ≤ 0.001), CKQ (F = 69.76, *p* < 0.001), CBC risk behavior (F = 4.62, *p* = 0.033), CBC adaptive behavior (F = 15.2, *p* < 0.001), CBC preventive behavior (F = 27.75, *p* < 0.001), but not the PHQ-4 (*F* = *1.63*, *p* = 0.204). The comparison of the refugees’ subsamples revealed significantly lower CKQ (*p* < 0.001, *T* = 20.21, df = 74), as well as significantly higher PVD (*p* = 0.008, *T* = − 2.73, df = 74) for Arabic speaking refugees. The increase in PVD of Arabic as compared to Farsi speaking refugees remained significant even when controlling for CKQ in an analysis of covariance (*p* = 0.004, *F* = 114.4, df = 1).

Additional linear regression analyses were conducted to identify the influence of PVD and CKQ on CBC risk, preventive, and adaptive behavior. CBC adaptive behavior was significantly predicted by the interaction between CKQ and group ($\beta$ =−0.286, SE = 0.167, *p* < 0.001), with low CKQ in the refugees’ group was associated with less adaptive behavior, and high CKQ in the German control sample being associated with higher adaptive behavior.

In addition, a high level of PVD was significantly predicted by low CKQ ($\beta$= − 0.239, SE = 0.021, *p* = 0.003).

Yet, there was no significant influence from CKQ on PHQ-4 ($\beta$=−0.044, SE = 0.016, *p* = 0.590). Also, there was no interaction between CKQ and group on PHQ-4 ($\beta$ =0.115, SE = 0.172, *p* = 0.244). However, there was a significant interaction between PHQ-4 and PVD, with a low PHQ-4 and high PVD predicting a higher preventive behavior ($\beta$ =− 0.190, SE = 0.056, *p* = 0.012). Based on the data from all participants, a higher level of CBC risk behavior was predicted by a reduced CKQ ($\beta$= − 0.418, SE = 0.022, *p* < 0.001).

## Discussion

We investigated the relationship between perceived vulnerability to disease and knowledge about COVID-19 and their influence on different styles of behavioral change and emotional distress. Moreover, we examined whether Farsi and Arabic speaking refugees reported a higher PVD than matched controls.

Consistent with our expectations, risk behavior and perceived vulnerability to disease were significantly larger in refugees than in matched controls. This is supported by findings that health care barriers for refugees might impede the development of preventive behavior [30]. In addition, refugees had significantly less knowledge about COVID-19, and showed less preventive and adaptive behavior. This is in line with findings that show less knowledge and poor health care access for migrants compared to non-migrants [31]. However, we did not find an elevated level of psychopathological distress in refugees. As there was a negative influence from knowledge on emotional distress, long-term effects on the psychological health still might be expected [4]. A possible explanation for this unexpected finding is that psychological distress during a pandemic is not only caused by knowledge or infection-related fears, but also by uncertainty of employment, care for elderly relatives, and social retrieval [32]. In addition, Miller et al. have found that in Afghan refugees with chronically increased levels of PTSD symptoms, high daily stressors paradoxically reduce the salience previous war-related experiences [33]. Yet, our findings show the tendency that a lower psychopathological distress predicts a higher preventive behavior. This is in line with recent findings that show milder ranges of depressive symptoms for persons with higher preventive behavior during the pandemic [34]. Therefore, preventive behavior might have stress-buffering function.

The lack of knowledge on COVID-19 in refugees might be explained by language problems and cultural barriers to the access of health information [2]. This impairment may be additionally increased by a lower educational level as reported in our refugee sample. However, it should be mentioned that Arabic speaking refugees had less knowledge about COVID-19 and a lower perceived vulnerability to disease. Due to the large outbreak and high death rates in early spring in Iran and Afghanistan, awareness was raised in the general population [35, 36]. Subsequently, Farsi speaking refugees might have been informed about COVID-19 through relatives living in Iran. On the other hand, infection numbers in Arabic regions have been moderate until now. Therefore, Arabic speaking refugees might be less informed through relatives in Syria et al. [37].

Generally, the PVD was significantly increased in the refugees’ sample. Yet, it is not clear whether a high PVD can be explained by the cultural background of the participants. Refugees share aspects of a social environment (e. g. postmigration stressors, low social participation) that might lead to a higher PVD [38]. Still, our findings are in line with Skolnick and Dzokoto [16], revealing that refugees have a higher PVD than matched controls with a different cultural background. This may indicate a sociocultural influence on the perception and cognitive processing of contagious viruses such as COVID-19, since the refugees of our sample originated from regions with higher numbers of contagious diseases (e.g. malaria or tuberculosis; 19). Increased experiences with pandemic diseases might increase the PVD [20], through a permanent activation of the behavioral immune system [39].

Furthermore, we could observe a negative relationship between knowledge and PVD in the refugee sample. Though it might be argued that PVD is largely influenced by knowledge, a higher PVD still could be found in refugees as compared to controls even after controlling for knowledge. This a further support for the findings of Skolnick and Dzokoto [16], that a high PVD is shaped by social (poverty, status) or cultural factors (e.g. high risk for infection).

There are several limitations of the presented study that should be considered. First, the small sample size of the refugee sample is responsible for a low statistical power. This only allows cautious interpretations of differences with respect to the educational level, knowledge and PVD. Furthermore, it is questionable whether the Farsi/Arabic speaking refugees of this study are representative for the respective population [40]. Due to the lack of familiarity with online surveys, a selective sample of refugees, for example such with a greater openness and willingness to stay informed, may have participated in this study. In terms of generalizability, another limitation may be the cultural heterogeneity of the refugee sample size. For example, we did not control sufficiently the influence of specific regions with higher infection rates and the respective ethnicities. This should be addressed in ongoing trials.

However, our findings reveal the importance of knowledge for preventive behavior. Refugees are considerably affected by missing information on COVID-19 and therefore especially exposed to possible harmful consequences. A further risk factor might be a contextually and culturally influenced PVD that leads to risk behavior. This emphasizes the need for culturally appropriate material to inform refugees about COVID-19.
